# Barriers and Facilitators of Smoking Cessation among Latinos Living with HIV: Perspectives from Key Leaders of Community-Based Organizations and Clinics

**DOI:** 10.3390/ijerph18073437

**Published:** 2021-03-26

**Authors:** Francisco Cartujano-Barrera, Michelle Lee D’Abundo, Evelyn Arana-Chicas, Surina Chock, Pamela Valera, Charles S. Kamen, Ana Paula Cupertino

**Affiliations:** 1 James P. Wilmot Cancer Institute, University of Rochester Medical Center, Rochester, NY 14642, USA; Evelyn_Arana@URMC.Rochester.edu (E.A.-C.); surinachock@gmail.com (S.C.); Charles_Kamen@URMC.Rochester.edu (C.S.K.); Paula_Cupertino@URMC.Rochester.edu (A.P.C.); 2 Department of Interprofessional Health Sciences and Health Administration, Seton Hall University, Nutley, NJ 07110, USA; michelle.dabundo@shu.edu; 3 Department of Urban-Global Public Health, Rutgers University, Newark, NJ 07102, USA; pv181@sph.rutgers.edu

**Keywords:** smoking, smoking cessation, Latinos, people living with HIV, HIV

## Abstract

The purpose of this study was to identify the perspectives from key leaders of community-based organizations (CBOs) and clinics serving people living with HIV on barriers and facilitators of smoking cessation among Latino smokers living with HIV. Semi-structured interviews were conducted in English and Spanish with 10 key leaders. Using a social ecological model, qualitative theoretical analysis was used to analyze the results. Participants identified barriers at the individual (e.g., low education level, HIV, and financial stress), interpersonal (e.g., language barriers, low social support), organizational (e.g., lack of smoking cessation resources and targeted interventions), community (e.g., HIV and mental health stigma), and policy (e.g., paperwork for insurance) level. Participants identified facilitators at the individual (e.g., high participation in trials, good medication adherence), interpersonal (e.g., no smoking in social circles), organizational (e.g., bilingual staff, culturally competent care), community (e.g., providing transportation, the coronavirus disease 2019 as an opportunity for smoking cessation), and policy level (e.g., existence of funding, comprehensive insurance programs). These results provide operational strategies to address smoking disparities among Latino smokers living with HIV. Further research is needed on how to integrate these perspectives into effective smoking cessation interventions.

## 1. Introduction

Tobacco use remains the leading preventable cause of disease and death among Latinos [1], the largest racial and ethnic minority group in the United States (U.S.) [2]. The ethnic terms Hispanic or Latino refer to a person of Cuban, Mexican, Puerto Rican, South or Central American, or other Spanish culture or origin regardless of race [2]. Of the more than 60 million Latinos who reside in the U.S., 9.8% are currently cigarette smokers [3]. The majority of Latinos experience multiple barriers to healthcare access and treatment that result in tobacco-related disparities. Compared to non-Hispanic Blacks and Whites, Latinos are less likely to receive advice to quit, participate in smoking cessation programs, and use pharmacotherapy for smoking cessation [4,5,6,7,8,9].

Decades of research demonstrate the efficacy of pharmacotherapy and counseling for smoking cessation [10,11,12]. However, as highlighted in the 2017 National Cancer Institute Tobacco Control monograph *A Sociological Approach to Addressing Tobacco-Related Health Disparities*, disparities in tobacco use, treatment outcomes, and disease burden persist, and elucidation of culturally and linguistically appropriate interventions are needed to advance tobacco cessation treatment among underserved, racial, and ethnic groups, including Latino smokers and especially among those living with HIV [13].

Latinos represent 16% of the U.S. population, but account for 25% of all new HIV infections [14]. Antiretroviral therapy has led to a decline in AIDS-related mortality and increased life expectancy among people living with HIV (PLWH) [15]. However, improved survival is tempered by alarming increases in non-AIDS-defining cancer, most notably lung cancer [16,17,18,19,20]. This increase is partly attributable to high smoking rates among PLWH (40%) compared to the general population (19%) [21,22]. Altekruse et al. showed that among PLWH, approximately half of smoking-related cancer, and 94% of lung cancer diagnoses, could potentially be prevented by quitting tobacco smoking [23]. Despite interest in quitting [24,25,26], cessation rates are relatively low among PLWH [27].

A particular subgroup of Latinos that faces tobacco-related disparities are Latinos living with HIV. In two nationally representative cross-sectional surveys, the prevalence of smoking among Latinos living with HIV was 35% [21]. In a cross-sectional survey conducted in New York City, 50% of smokers living with HIV were Latinos [28]. Despite these alarming smoking rates, to our knowledge, only one randomized clinical trial focused on Latino smokers living with HIV has been conducted, which indicated that an in-person counseling intervention does not improve cessation outcomes compared to brief advice (8% vs. 11% cessation at 6 months, *p* = 0.92) [29].

Because of the paucity of research on tobacco cessation treatment among Latino smokers living with HIV and their relatively low cessation rate, it is crucial to understand the barriers and facilitators of smoking cessation among this population. These findings could guide the development of culturally and linguistically appropriate interventions for smoking cessation among Latinos living with HIV. As such, the purpose of this study was to identify the perspectives from key leaders of community-based organizations (CBOs) and clinics serving PLWH on barriers and facilitators of smoking cessation among this high-priority population.

## 2. Materials and Methods

### 2.1. Study Design

This qualitative study consisted of semi-structured interviews in English or Spanish with key leaders of CBOs and clinics serving PLWH. Study procedures were approved and monitored by Hackensack University Medical Center’s Institutional Review Board (protocol number Pro2020-0089).

### 2.2. Participants

Between April and June 2020, 10 key leaders of CBOs and clinics serving PLWH were recruited to participate in this study. Individuals were eligible if they were ≥ 21 years old, directly worked with PLWH, spoke English and/or Spanish, and were interested in participating in a 60 min interview. Participants received a $20 gift card as a compensation for their time and effort.

### 2.3. Recruitment, Eligibility, and Consent

Purposeful sampling method was used to identify key leaders of HIV care with different professional perspectives (e.g., medical doctors, nurses, physician assistants, case managers, researchers, and managers). Individuals who were responsible for developing, implementing, and/or sustaining efforts for HIV care and treatment were considered key leaders [30]. A snowball sampling method was also employed by asking participants to refer to additional individuals and/or organizations to take part in this study. Individuals were identified from CBOs and clinics serving PLWH in the states of New Jersey and New York. Consistent with the Centers for Disease Control and Prevention, CBOs were defined as social service agencies, nonprofit organizations, and formal and informal community groups, such as neighborhood groups or recreational or special-interest clubs, which work at the local level to meet community needs [31]. Potential participants were initially contacted through an email, which included details about the purpose of the study and directed them to contact the first author regarding their interest in participation. The email was available in English and Spanish. If responding individuals were interested in the study, the first author scheduled a phone call to further discuss the study and conduct the eligibility assessment. The eligibility assessment was conducted in English or Spanish, as preferred by the individual.

All participants were provided with an electronic copy of the study information sheet, providing detailed information about the purpose of the study, potential risks and benefits, incentives, study procedures, voluntary participation and the right to withdraw, confidentiality, data management, and contact information of the study team. The study information sheet was available in English and Spanish. The first author reviewed the information sheet with participants and provided them with the opportunity to ask questions prior to agreeing to participate in the study. Verbal consent was obtained from the participants over the phone prior to participation in the study.

### 2.4. Data Collection

Prior to the interview, participants completed a sociodemographic survey that collected measures on age, gender, race, ethnicity, years working with PLWH, current position, number of people their organization serves per month, and percent of Latino clients their organization serves per month. The survey was available in English and Spanish.

Immediately after the sociodemographic survey, a semi-structured interview was conducted. An interview guide with a list of open-ended questions was used to facilitate the interviews. The social ecological model (SEM) was used as a framework for interview guide development, in order to understand different levels of barriers and facilitators of smoking cessation among Latinos living with HIV [32]. The SEM considers the multiple levels of factors that influence health and health behaviors, at the level of the individual, interpersonal, organizational, community, and public policy [33]. Individual-level factors are the characteristics of an individual, including knowledge, attitudes, and skills [34,35]. Interpersonal factors are social support systems and social networks, including family, friendship, and work group networks [34,35]. Organizational-level factors are institutional characteristics associated with organizations and their formal/informal operation, as well as rules and regulations [34,35]. Community-level factors include the characteristics related to the location in the community, culture, neighborhood associations, built environment, and transportation [34,35]. Policy-level factors are local, national, and/or international laws and policies that serve as a mediating structure to connect people and the larger social environment to make healthy choices [34,35]. Table 1 shows examples of the interview questions by SEM level.

The interviews were conducted in English or Spanish, as preferred by the participant, by the first author. All interviews were audio-taped with participants’ permission and subsequently transcribed verbatim in the language in which they were conducted to preserve meaning.

### 2.5. Data Analysis

For the sociodemographic survey, means and frequencies were calculated. Participants’ years of experience working with PLWH were categorized in (1) 5 years or less, (2) more than 5 years but less than 10 years, (3) between 10 and 20 years, and (4) over 20 years. For the interviews, qualitative theoretical analysis was used to identify, analyze, and report themes within the data [36]. The first and fourth author, who are fluent in English and Spanish, first coded the first five transcripts independently. An iterative process was employed to achieve consensus between the two sets of codes, create a coding scheme, and develop a codebook that included details about agreed-upon code definitions. New codes were added as needed to the coding scheme until no new themes emerged with successive interviews [37]. Once the codebook was developed, quotes in Spanish were translated into English by the first author and reviewed by the fourth author. The second author supervised the qualitative theoretical analysis and provided in-depth feedback in the codebook development. All authors agreed on the final themes and sub-themes.

## 3. Results

Ten key leaders from five CBOs and three clinics participated in the interviews. Table 2 describes the participants’ type of organization where they work and years of experience working with PLWH. Table 3 describes the sociodemographic characteristics of the participants. The participants’ mean age was 42 years (SD 11.4), 70% were male, 40% identified as White, 30% identified as African American, 40% identified as Hispanic or Latino, and 80% preferred to complete the survey and interview in English. Regarding their current position, the sample consisted of two medical doctors, a nurse practitioner, a physician assistant, three case managers, one research coordinator, and one manager. On average, participants had 15 years of experience working with PLWH (SD 11.2). On average, organizations served 535 PLWH per month (SD 745.7; Range 50–2500), of which 33% were Latinos (SD 11.8).

### 3.1. Individual Level

Key leaders of CBOs and clinics perceived 10 individual barriers of smoking cessation among Latinos living with HIV: (1) low education level, (2) financial stress, (3) HIV stress, (4) minimal understanding of the benefits of quitting smoking, (5) minimal interest in smoking cessation counseling, (6) minimal interest in smoking cessation medications, (7) smoking and mood management, (8) smoking and weight management, (9) alcoholism and other substance use disorders, and (10) low self-worth. Moreover, four individual facilitators were identified: (1) participation in clinical trials, (2) good medication adherence, (3) understanding of the cost-saving benefits of quitting, and (4) physical activity for smoking cessation. Select quotes from participants are shown below.

#### 3.1.1. Barriers

*Low education level.* Three participants noted that low education level was a barrier to providing healthcare:

“The people who come here [to the clinic] from the Latino community, many of them do not have an education beyond high school. Here [New York] we have a very large population of people from Puerto Rico. But it’s people who lived in the fields of Puerto Rico. So, when they get here [New York], they are not always able to understand their health needs and make healthy decisions… Unfortunately, in the fields people often don’t have access to complete education, not even to finish high school.”

Participant 5

*Financial stress*. Nine participants described Latino smokers living with HIV experience anxiety and/or worry as the result of economic insecurity. This economic scarcity precluded Latino smokers living with HIV to meet basic needs related to nutrition, housing, and healthcare:

“There are a lot of people within the Latino community that don’t have a lot of financial resources. This is a big stressor for them.”

Participant 2

One participant noted that, even though tobacco spending may exacerbate financial hardship, smoking was a coping mechanism to financial stress among Latino smokers living with HIV:

“They [Latino clients] face lots of financial stressors, which you think seems kind of counterintuitive, right? But that’s what happens, you know, you are financially stressed, you spend money to buy cigarettes, right? But that’s the way it happens.”

Participant 3

Although some Latino smokers living with HIV relied on state-subsidized housing programs, activities such as filling out forms to comply to housing programs rules and regulations were stressful, as described by one participant:

“They [Latino clients] worry because of their Department of Housing [and Urban Development] forms [for public housing]. They do it every month, but they always get stressed. They need to complete it and bring it in time. They have a lot of financial stress.”

Participant 5

*HIV Stress*. Three participants noted that Latinos living with HIV have the perception that HIV is a terminal disease rather than a chronic illness:

“Although people [living] with HIV live longer now, a lot of people think that’s not true. A lot of Latinos still think that ‘Oh, once you have HIV, your life is over’ as opposed to think ‘No, there’s a lot that I can do to survive.’… It’s a matter of educating the community. They need to know that having HIV does not equal dying, it just means that they will need to be more responsible with their health and that includes quitting smoking.”

Participant 1

*Minimal understanding of the benefits of quitting smoking*. Eight participants described that Latino smokers living with HIV perceive minimal understanding of the benefits of quitting smoking. One participant noted that as long as Latino smokers living with HIV do not have a lung problem, Latino clients will not consider quitting smoking:

“What I’ve noticed is that if they [Latino clients] don’t have a lung problem or a symptom, then they’re going to think they are fine.”

Participant 5

Five participants noted that Latino smokers living with HIV do not seem to be aware of the specific benefits to quitting smoking for PLWH:

“A lot of Latinos [living with HIV] are not aware of the specific issues or I guess the heightened risk of health issues that come with the commodity of smoking and being HIV positive. So, a lot of folks I think are like ‘Oh yeah, of course I know that it’s not good for my lungs, sure.’ But I don’t think that a lot of folks recognize that, for example, that there’s a heightened risk of heart disease and heart issues, just from being diagnosed with HIV. And then you put smoking on top of that, that’s compounding that issue. So, I think that people are aware of the general issues but are not well aware of the specifics when it comes to smoking while being HIV positive.”

Participant 6

One participant described that Latino smokers living with HIV do not seem to understand the impact that smoking has with HIV medications:

“I don’t think they [Latino clients] understand the effects that smoking has with the [HIV] medications... I don’t think they understand. Even when we tell them, they look at us like ‘What are we talking about? How does my smoking affect my medication?’”

Participant 8

*Minimal interest in smoking cessation counseling.* Seven participants noted that Latino smokers living with HIV have minimal interest in smoking cessation counseling. Participants described that Latino smokers living with HIV perceive smoking cessation counseling as unnecessary or overly sentimental (e.g., “corny”):

“There are times that we referred them [Latino clients] to a counselor [for smoking cessation], right? Very few accept the counselor to tell you the truth… They always tell me they don’t believe in it. ‘It’s all about willpower,’ they say. I think that the few Latinos who accept counseling just say ‘Yes’ to make us happy. Just to make us happy. I don’t think they say ‘Yes’ because they really want to.”

Participant 5

“A lot of times the Latino folks don’t wanna go there [to counseling]. I have heard things like ‘I don’t really have that big of a problem.’ Some others are just like “That is corny, I’m not gonna do that.’”

Participant 6

One participant described that even though Latino smokers living with HIV were referred to smoking cessation counseling, attendance to in-person appointments was low:

“We have tried for other providers to schedule them [Latino smokers] with me for a smoking cessation visit… But I found attendance to those appointments was very poor. Very poor… Even if I tried to like call them and bring them back, it’s just not… It was very poor… I think it’s just the idea of having one more medical appointment.”

Participant 3

*Minimal interest in smoking cessation medication.* Three participants noted that Latino smokers living with HIV have minimal interest in smoking cessation medications. As described by one participant, this could be due to Latinos living with HIV feeling overwhelmed with already taking numerous medications:

“There’s the idea of ‘I don’t want more medication’ or ‘If I’m going to do it [quit smoking], I wanna do it on my own,’ which are valid reasons. A lot of our patients don’t want one more medication… It’s kind of more that type of pill fatigue… Although even, even people who are just taking like two pills a day are like ‘I don’t want to add more pills,’ and I’m like ‘You’re on two pills a day, that’s not very much.’”

Participant 3

*Smoking and mood management.* Seven participants perceived that Latino smokers living with HIV use cigarettes to manage stress and anxiety:

“I think it [smoking] has to do with the fact that they [Latino clients] feel oppressed and smoking is the way that they release whatever anxiety or whatever stressors they have in their life.”

Participant 1

“Smoking is something that they [Latino clients] hold, they have control over. It’s something that can help regulate their mood. I believe that they find things like smoking as something that helps control their moods.”

Participant 2

“Some of my Latino clients are just like ‘My go to is smoking and when I’m so stressed, I can’t remember of anything else, so I just go and smoke a cigarette.’”

Participant 6

*Smoking and weight management.* Five participants described that Latino smokers living with HIV voice concerns about smoking and weight management. As noted by one participant, Latino smokers living with HIV have the perception that smoking is a weight control method:

“Another thing that I have heard is that smoking helps them [Latino clients] be in shape, like in their healthy weight.”

Participant 1

Moreover, participants noted that Latino smokers living with HIV have concerns about weight gain when quitting smoking. These concerns were based on previous quit attempts:

“The [Latino] clients that tell you the reasons for them not wanting to quit smoking, always give us excuses. For example, ‘Every time I quit smoking, I get very anxious about something else, then I start eating more and gain weight.’ They don’t want that.”

Participant 5

“They [Latino clients] don’t want to stop [smoking] because they begin to eat, they begin to gain weight. They are substituting food for cigarettes.”

Participant 9

*Presence of alcoholism and other substance use disorders.* Seven participants noted that alcohol, marijuana, cocaine, and other substances were commonly used among Latino smokers living with HIV and were perceived as barriers to smoking cessation:

“Another thing that I think it’s making them [Latino clients] hard to quit [smoking] is that they don’t just smoke regular cigarette smoke, they also use marijuana.”

Participant 1

“A number of Latino patients have a history of drug use, whether they are currently using or not. And for a lot of our clients, smoking, which of course is not healthy or great for them, is kind of the vice they picked up when they gave up another drug use. So, I think because of that, sometimes case managers are less likely to kind of push the [smoking cessation] issue.”

Participant 6

“I think that the biggest contributor to not quitting smoking is substance abuse. So, people who are really active substance users, particularly cocaine users, have a hard time [quitting].”

Participant 7

*Low self-worth.* One participant noted that in order to enhance smoking cessation treatment among Latino smokers living with HIV, self-worth should be recognized and strengthened:

“For them [Latino clients] to quit [smoking], we need the input of mental health professionals. That would help. Because the mental health professional will help with the fact that they are worth something and help them envision what they’re worth and help them plan for the future of being who they really are.”

Participant 2

#### 3.1.2. Facilitators

*Participation in clinical trials.* Two participants noted that PLWH are willing to participate in clinical trials:

“Well, you need to know that our HIV patients are very open to participating in clinical trials because they’ve been kind of exposed to it. In the HIV world, we have lots of types of research things going on.”

Participant 3

*Good medication adherence.* Four participants described good adherence to HIV medications among Latinos living with HIV:

“I feel that that they [Latino clients] do a pretty good job taking medicines. I get here and there but I wouldn’t say that across the board there’s a barrier to taking their [HIV] medication.”

Participant 3

“Ninety-five percent of the time, they [Latino clients] are adhering to their [HIV] medications. They understand how HIV can mean death and that they… I think they’re motivated by that, to avoid that. They take their medications.”

Participant 4

“Most of my clients that I have that are Latino have been adhering to their [HIV] medication as well as their doctor’s appointments.”

Participant 9

*Understanding of the cost-saving benefits of quitting.* One participant noted that elucidating the economic benefits of quitting smoking has been an effective strategy for smoking cessation among Latino smokers living with HIV:

“I know some Latino clients who sat down and did a budget one year and realized that between he and his wife, they were both spending eleven thousand dollars a year on cigarettes… So, they were thinking that in two years they could buy a car. So, you know, that was their motivation to do a [nicotine] patch [for smoking cessation].”

Participant 2

*Physical activity for smoking cessation.* Three participants described that physical activity is an effective strategy for smoking cessation among Latino smokers living with HIV:

“Something that has helped my Latino clients quit [smoking] is to add exercise into their life.”

Participant 2

“Exercising. People start feeling good when exercising. So that’s one thing that I believe will work [to quit smoking], you know? They [Latino clients] need to have positive activities such as, you know, waking, running…”

Participant 7

### 3.2. Interpersonal Level

Participants identified three interpersonal barriers of smoking cessation among Latinos living with HIV: (1) language barriers, (2) smoking in social circles, and (3) low social support. Furthermore, no smoking in social circles was identified as an interpersonal facilitator of smoking cessation. Select quotes from participants are shown below.

#### 3.2.1. Barriers

*Language barriers*. Seven participants noted that limited English proficiency was a barrier to delivering HIV care and treatment. As exemplified below, limited English proficiency was a barrier to effective communication, accessing services, and filling prescriptions:

“Sometimes they [Latino clients] only speak Spanish… Sometimes they’re not comfortable. They’re not comfortable if they only speak Spanish… And even if they speak English, if Spanish is their first language, there’s a lack of communication. There’s a lack of being able to communicate effectively.”

Participant 1

“I think the language barrier is a huge barrier, you know. It’s harder to make your needs known if you can’t communicate with someone. Whether it be calling the pharmacy for refills or calling and scheduling an appointment.”

Participant 3

“They [Latino clients] can be frustrated if they try speaking English and can’t be understood. They immediately feel frustrated and find in a roadblock if they can’t communicate… I find that happens often, not only in the clinic, but in the pharmacies as well. We have a pharmacy and they can… They can be a little bit frustrated when their needs can’t be communicated and they can’t understand. So, that’s difficult in terms of language and communication.”

Participant 4

“If they [Latino clients] only speak Spanish, they might not have somebody that could translate for them or they might think that they [the smoking cessation counselors] don’t speak Spanish… I think that discourages them from reaching out.”

Participant 8

*Smoking in social circles.* Five participants observed that Latino smokers living with HIV who are surrounded by friends, family members, and/or roommates who smoke have a hard time quitting smoking:

“I’ve seen [Latino] clients that work so hard [to quit smoking] but they’re always around all of their friends, their relatives, and they all smoke... They’re doomed because no matter where they go out, there’s somebody else that’s reminding them that there’s a possibility of smoking... There’s a social component.”

Participant 2

“If they [Latino clients] are in a household where a lot of people smoke or in a culture where smoking is seen as something desirable or something that is normal to do, that is kind of a barrier [for smoking cessation]… It can be very difficult to stop [smoking] because, you know, they’re surrounded by it. They’re like ‘Well, I’m breathing in the secondhand smoke anyways so I’m gonna smoke’ or ‘You know, if I tell myself I’m not gonna smoke, then I don’t buy any cigarettes. But then I know there’s always someone in my house that smokes and I’ll go ahead and smoke.’”

Participant 6

*Low social support.* One participant noted, based on conversations with Latino smokers living with HIV, that low social support, specifically lack of family engagement, was a barrier for smoking cessation:

“From conversations that I’ve had with [Latino] participants is that loneliness is a stressor… It’s heartbreaking, because you hear them talk about their families, that their families don’t care about them. They need shelters, so cigarettes it’s kinda like a crutch for them, to get them by the day.”

Participant 8

#### 3.2.2. Facilitators

*No smoking in social circles.* One participant noted that living in a smoke-free home was a facilitator for smoking cessation:

“I’ve notice that Latinos who are hanging around others that don’t smoke, they are more likely to quit.”

Participant 2

### 3.3. Organizational Level

Four organizational barriers were identified by participants: (1) lack of smoking cessation resources, (2) lack of targeted interventions for Latinos, (3) healthcare navigation difficulties, and (4) low quality of translation services. Six organizational facilitators were identified: (1) first HIV care visit for smoking cessation, (2) bilingual staff, (3) accommodation for non-English patients, (4) culturally competent care, (5) comprehensive outreach activities, and (6) high engagement from the community. Select quotes from participants are shown below.

#### 3.3.1. Barriers

*Lack of smoking cessation resources.* Three participants described the lack of printed resources for smoking cessation in the organizations where they work:

“We are not really well stocked with those educational pamphlets [for smoking cessation].”

Participant 4

*Lack of targeted interventions for Latinos.* Two participants noted the lack of smoking cessation interventions tailored to Latino smokers living with HIV:

“I’m sure they [Latino clients] have heard about the national campaigns [for smoking cessation]. But maybe we didn’t let them know in a way that resonated with them… Maybe the campaigns weren’t appealing to Latinos… Maybe they were not even available in Spanish…”

Participant 1

*Healthcare navigation difficulties.* Three participants described that Latinos living with HIV, particularly foreign-born Latinos, have healthcare navigation difficulties:

“The lack of understanding of the system and knowing how to navigate the whole medical system in the United States is a barrier, especially if they are coming from a different country. They [Latino clients] may not be well familiarized with the insurance, how to get it, and how to end up paying health insurance.”

Participant 4

*Low quality of translation services.* Two participants noted that, although their organizations have translation services (e.g., live or remote interpreters) available for Spanish speaking clients, services were of low quality:

“We have a Spanish interpreter. We bring her in if we need her help to translate Spanish. It honestly slows the appointment down. You have to pause, wait for the interpreter to translate what you have said and then you have to wait for the interpreter to translate what the patient said. So, there is always a little bit of delay. It’s a very slow process.”

Participant 2

“You can’t always get an in-person interpreter. Then you have to use the CyraCom^®®^ interpreter using an iPad^®®^ which is very less than ideal. Also, the quality of the CyraCom^®®^ interpreters is sometimes absolutely horrendous.”

Participant 3

#### 3.3.2. Facilitators

*First HIV care visit for smoking cessation.* One participant described that the first medical appointment to receive HIV treatment was an important moment to start having a conversation on smoking cessation with Latino smokers living with HIV:

“In the first appointment you always ask: Do you smoke? We also ask about the frequency, the amount… And we offer things on that first appointment... They [Latino clients] are asked if they have tried to quit smoking. All routine questions are asked when you are doing the examination… I always think that the very first appointment to receive HIV treatment is the most important. It’s a powerful appointment. If we offer them help to quit smoking, they usually always say yes. Of course, there are not always ready but that first visit is key. Even when they say, ‘Well, I will think about it,’ they always mention it at the end of the appointment. We should use more and more that first visit to invite them to quit smoking. We should start offering medications, right? They want to quit [smoking], they just don’t know how to do it.”

Participant 5

*Bilingual staff.* Seven participants noted that their organizations have bilingual staff (English and Spanish). This was identified as a facilitator for HIV care among Latinos:

“About three years ago, we… We increased our capacity to serve Latinos by hiring… I would say that 90% of all of our new staff are Latinos or bilingual. All the outreach team, except for one person, speak Spanish.”

Participant 2

“We have care managers that speak the Spanish language so it’s very user friendly for the Latino population to come to [Name of Organization] and get their care there.”

Participant 4

“On many occasions what we do with them [Latino clients] is that I do the translations. I am one of the a few healthcare providers who speak Spanish, right? If I am here, then we don’t need to use an interpreter and the person is assigned to me.”

Participant 5

“So, we do have a couple folks on the team who are bilingual and who kind of specialize in different things. We have two folks who do HIV case management and focus on I guess like general HIV information, and then we have someone else who is bilingual who focuses more broadly on other health concerns, such as other STIs and things like that.”

Participant 6

“We at least have one or two people that speak Spanish at every regional office that we have.”

Participant 10

*Accommodation for non-English patients.* Two participants described that their organizations have a formal process in place to ensure that interpreters were available for non-English speaking clients in advance of their visits:

“If we know that one person that can’t speak English comes in advance, then the request is made for an interpreter. There are interpreter services here in [name of organization] so the request is made and the interpreter comes.”

Participant 5

*Culturally competent care.* Two participants noted their organization recognized that Latinos come from a variety of countries and that their staff were representative of this diversity to facilitate rapport with clients:

“I think we do a good job by hiring quality people who care about the community. Because clients are not just Puerto Ricans, they’re Dominicans, they’re Mexicans... Having members that can related to them, in the frontline contacts… That’s something [Name of Organization] wants.”

Participant 2

*Comprehensive outreach activities.* Six participants noted that their organization have comprehensive outreach activities for Latinos. Outreach activities included going into the neighborhoods, nightclubs, and faith-based organizations with high concentration of Latinos:

“We go to the Latino neighborhoods and we try to engage them. We provide lots of health services that are not necessarily HIV-related. Just to let them know that we’re in the area and that we’re people that can be trusted. We have learned that being upfront about HIV testing is a little scary… People don’t usually talk to us if we only do HIV testing. We need to offer more services.”

Participant 1

“We go to areas where Latinos are. We go to clubs on Thursday nights… Thursday nights are Latino nights… We go there.”

Participant 2

“Oftentimes things that we’ve done with some of our faith-based organizations is that we often go in and do something more generally related to HIV/AIDS awareness. For example, we’ve gone to a couple different houses of worship for like World AIDS Day to talk about HIV/AIDS. For that we’ve also come to some schools, varying from high schools to community colleges to talk about HIV/AIDS and sexually transmitted infections as well.”

Participant 6

“We have a department that goes out into the street to reach Latino clients by knocking on their doors or trying to find to them where we know they may hang out at. We have community health workers doing an outreach service for our program.”

Participant 9

“So, for the Latino community, when it comes to doing outreach we mostly go with the most Latino densely populated areas. We usually go to those counties if we decide to target the Latino community.”

Participant 10

*High engagement from the community.* Three participants described that their organization included people from the community to build and implement their operational plan:

“We ask people from the community to volunteer and we ask them to be part of our programs. We include, to the best of our ability, people who are gonna be part of the process and make decisions as well. We have a Latina that has joined our board that helps make, you know, decisions on the executive level as well.”

Participant 2

### 3.4. Community Level

Key leaders of CBOs and clinics identified HIV and mental health stigma as community barriers of smoking cessation among Latinos living with HIV. Furthermore, providing transportation and knowledge on COVID-19 as an opportunity for smoking cessation were identified as community facilitators. Select quotes from participants are shown below.

#### 3.4.1. Barriers

*HIV stigma.* HIV-related stigma in the Latino community was identified as a barrier for HIV care, including uptake of smoking cessation services offered at their organizations. As noted by one participant, Latinos, particularly foreign-born Latinos, wrongly identify HIV as a “gay” disease:

“People used to say that HIV was “gay-related”. Has that mentality changed for people? Today? In 2020? I am not sure… Especially among Latinos… It goes back in like years and years of like bad mentality, or bad education, you know? Are we educating the community? Yes, since high school. But with Latinos is a little challenging because if they come to this country, if they migrate here, it’s like every… They may come to the U.S. with those ideas in their heads… It’s hard changing someone’s ideas. That is a big challenge.”

Participant 1

Another participant noted that the Latino community is relatively small and that many Latinos were concerned that other members of the community would know their HIV status:

“The Latino community here is large but not so large, true? We know a lot of people in our community. Within the Latino community, there is still a lot of stigma regarding HIV. Some people will suddenly think, ‘I don’t want other Latinos to know my status.’”

Participant 5

*Mental health stigma*. Five participants described that Latino smokers living with HIV face mental health stigma, preventing them from getting help:

“They [Latino clients] don’t like to talk about their feelings. Do they really talk about depression? Do they talk about things that do with their mental health? They don’t. I think that mental health is right there buried in the back. Even the words ‘mental health.’ Latinos immediately think, ‘esos están locos [they are crazy].’”

Participant 1

“Mental health... I do feel that the Latino population is not as open to engaging in mental health treatment… I think some of it could be because they don’t want people to think they are crazy.”

Participant 3

“Mental health is definitely an issue that I feel like is not well addressed among our Latino clients... I think that in the Latino communities we serve, there is a lot of stigma around mental health. When we discuss the need for taking care of mental health, they would have this kind of cultural thing of ‘I’m not crazy. I don’t need that. I don’t want to talk about that.’ Even if we, you know, have conversations about taking care of your mental health isn’t saying you’re crazy, it’s just we all have brains and we should all make sure they’re healthy. But there’s still kind of oftentimes in the Latino community a cultural block that we see.”

Participant 6

#### 3.4.2. Facilitators

*Providing transportation.* Two participants noted that providing transportation to their Latino clients enhanced adherence to medical appointments:

“So, as long as we help them [Latino clients] with transportation, they are willing to attend their [medical] appointments.”

Participant 5

“Whenever we can provide Uber or Lyft for clients, they [Latino clients] are really thankful. This is a way to build trust and make sure they adhere to their [HIV] treatment.”

Participant 6

*COVID-19 for smoking cessation.* Three participants noted that the COVID-19 pandemic brought forth new opportunities to promote smoking cessation among PLWH:

“The situation that we’re having with the coronavirus [COVID-19] outbreak is, is something, that I think brings many… You can use this to help patients be more motivated to quit smoking. If you point out to them, if you educate them that those people who died from coronavirus usually are the ones that have lung disease and who have other medical diseases and that they smoke, that is something that may educate them and may convince them that smoking is not good and will lead to them getting sick and maybe being hospitalized.”

Participant 4

“I think in being locked at home [because of COVID-19], I think has kept a few of them [Latino clients] from smoking as much as they normally would smoke. Because they are not traveling as much, and they are not dealing with other people who smoke.”

Participant 8

### 3.5. Policy Level

At the policy level, participants identified the paperwork for insurance as a barrier. Existence of funding, comprehensive insurance programs, and smoke-free policies were identified as facilitators. Select quotes from participants are shown below.

#### 3.5.1. Barriers

*Paperwork for insurance.* One participant noted that, although PLWH qualify for programs like the AIDS Drug Assistance Program, Latinos living with HIV experience difficulties enrolling in health plans:

“They [Latino clients] have insurance issues in that, you know, they might not fill out their paperwork so they’re on and off, and on and off, and on and off insurance. But it’s not that we can’t get it for you, it’s just whether you keep it because you do what you need to do to keep it.”

Participant 3

#### 3.5.2. Facilitators

*Existence of funding.* Two participants described the existence of funding for HIV care as a facilitator to better serve PLWH:

“We have a grant care management system… We have AIDS Institute funding, which comes from the New York State Department of Health. We have several care managers that would directly assist patients with any issues that may arise in terms of their medical care or navigating the health system.”

Participant 4

*Comprehensive insurance programs.* Six participants noted the existence of comprehensive insurance programs for PLWH was a facilitator for smoking cessation. Participants described that insurance programs for PLWH cover nicotine replacement therapies (e.g., nicotine patches, gum, lozenges, nasal spray, and inhaler) and varenicline for smoking cessation:

“They [Latino clients] would need to get insurance, in order to get their [smoking cessation] medications… We would have them see a healthcare navigator. Then they will get some kind of Medicaid, if they qualify for Medicaid or, HIV patients also can qualify for something called ADAPs, AIDS Drug Assistance Programs… If they qualify for ADAPs they can get those type of nicotine replacement products. I’ve never really had any rejected nicotine replacement products. It’s always been covered, in my experience.”

Participant 4

“So before, if you had somebody [a client] who was like hit or miss, hit or miss, kind of doing it [quitting smoking] but not really doing it, then maybe after six months they were ready to do it, and then you couldn’t get the medications [for smoking cessation] for them. That is no longer the case. Now they can get it for forever. If they wanna be on the nicotine replacement for forever, they could be on the nicotine replacement for forever. And way back, when Chantix^®®^ might not have been covered by every insurance… That too has pretty much changed. ADAP is the AIDS Drug Assistance Program. It’s a federally funded program but then given to the state, and then the state controls its. New York state’s ADAP is very generous… So now on ADAP, Chantix^®®^, nasal spray, and air inhaler are all covered.”

Participant 3

One participant noted that insurance programs for PLWH cover all patients, regardless of their immigration status:

“Most of the undocumented clients think that just because they’re undocumented, that they cannot get any health benefits, which in this case is not true. Every HIV-positive client gets health insurance, regardless of their immigration status.”

Participant 10

*Smoke-free policies.* One participant described that the existence of smoke-free policies was a facilitator for smoking cessation:

“I’ve seen that they [Latino clients] cut down the smoking when they are not going out to bars to drink, to socialize with their friends… They have less and less of a need, in my opinion, to smoke.”

Participant 2

## 4. Discussion

In the present qualitative study, semi-structured interviews were conducted with key leaders of CBOs and clinics serving PLWH to understand barriers and facilitators of smoking cessation among Latinos living with HIV. Their perspectives are key as they provide operational strategies to address smoking disparities among Latino smokers living with HIV. The study was framed under a SEM framework, an approach that facilitated the understanding of barriers and facilitators by studying the individual, interpersonal, organization, community, and public policy level. The findings are important as they may help healthcare providers and researchers to develop or adapt existing smoking cessation interventions to better meet the needs of this particular population.

The results of this study confirm and extend prior research conducted to understand barriers and facilitators of smoking cessation among Latinos. Our results support individual (e.g., smoking and mood management), interpersonal (e.g., language barriers and smoking in the social circle), and organizational (e.g., lack of targeted interventions) barriers previously identified by Carter-Pokras et al. [38]. Our findings also support the minimal interest in smoking cessation counseling and medications among Latinos described by Zinser et al. [39]. However, studies conducted by Cartujano-Barrera et al. show that, when offered in a culturally and linguistically appropriate manner, Latinos have high interest and utilization of smoking cessation behavioral interventions and pharmacotherapy [40,41,42]. Additionally, Shadel et al. conducted a qualitative study to enhance nicotine patch adherence among Latino smokers living with HIV [43]. Future smoking cessation interventions for Latinos living with HIV should take into consideration their findings. Lastly, consistent with Piñeiro et al., low education level, financial stress, and language barriers were identified as barriers of smoking cessation among Latinos [44].

Despite the support of previously identified barriers of smoking cessation among Latinos, this study shows that Latino smokers living with HIV encounter unique barriers at the individual level. As described by Martinez et al., being diagnosed with HIV is a traumatic experience for the great majority of Latinos, posing considerable stress related to HIV such as HIV-related stigma, disclosure concerns, antiretroviral treatment, and physical changes [45]. As noted by participants in this study, HIV-related stress and stigma are two major barriers to smoking cessation among Latino smokers living with HIV. Smoking cessation interventions for Latino smokers living with HIV should incorporate mental health and stress management as part of treatment. Furthermore, as described by one participant, Latino smokers living with HIV may experience the burden of multiple medical appointments. Text-messaging interventions for smoking cessation are an effective alternative to repeated clinic visits prompting behavior change and supporting adherence to treatment [46]. Moreover, text messaging interventions are easy to understand, available anywhere at any time, scalable to larger populations, and can incorporate real time-delivery assistance [46,47,48]. Cartujano-Barrera et al. have shown that a culturally and linguistically appropriate smoking cessation text messaging intervention for Latinos is well-accepted by participants, generates high satisfaction, significantly increases self-efficacy, produces high therapeutic alliance, and results in noteworthy cessation rates at the end of treatment [40,41,42]. However, this intervention has not been formally tested among Latinos living with HIV. As evidence shows that PLWH are less successful in quitting smoking than individuals without HIV [27], an adaptation of this intervention is needed to provide more intense and tailored behavioral support to the unique needs of PLWH.

Another unique individual barrier, identified by key leaders of CBOs and clinics, was Latinos’ minimal understanding of the benefits of quitting smoking. It is important to note that there is a possibility that efforts to educate Latinos living with HIV on the benefits of quitting smoking have not been adapted to their educational level, cultural preferences, and/or language of preference. Another possible explanation to this important barrier is that the stigma of living with HIV is so salient in the lives of Latino smokers that quitting smoking is not a priority for them. Delivering appropriate, sensitive, and effective educational information and engaging unmotivated smokers to move toward quitting is a public health priority to advance smoking cessation among Latinos living with HIV.

Key leaders of CBOs and clinics discussed that low social support seems to be an interpersonal barrier for smoking cessation among Latinos living with HIV. Social support plays a critical role as interpersonal relationships motivate behavioral changes [49]. De Dios et al., showed that greater social support network contact among smokers living with HIV was associated with higher levels of smoking cessation treatment adherence, and greater treatment adherence was positively correlated with smoking cessation [50]. However, as noted by De Dios et al., interpretation of the role of social support in smoking cessation treatment adherence and outcomes among PLWH require cautious and thoughtful consideration as social support domains may have a more distinct function among PLWH compared to the general population [51]. Another interpersonal barrier identified by participants was smoking in social circles. Social networks affect a variety of health behaviors, including smoking [52]. The mechanisms by which social networks have been found to influence health behaviors include social control, social norms, peer role modeling, and social support [53]. As noted by Twyman et al., social networks with a high prevalence of smoking, social acceptability of smoking, and a lack of social support for quitting smoking are barriers to smoking cessation [54]. Future research should examine how to address both low social support and smoking in social circles among Latino smokers living with HIV.

Two important community barriers of smoking cessation among Latinos living with HIV described by participants were mental health and HIV stigma. It is important to note that PLWH are at a higher risk for mental health disorders [55,56]. Research demonstrates that depression is associated with poor adherence to medical regimens, including smoking cessation treatment [57,58]. As Latinos are more likely to be at risk for depression than Whites and are less likely to obtain appropriate care [59,60], they may be at higher risk for smoking cessation treatment nonadherence. It is imperative that future smoking cessation interventions for Latinos living with HIV address mental health care. Regarding HIV stigma, our findings are consistent with studies showing that the cultural value of machismo among Latinos creates stigma associated with HIV and homosexuality [61,62]. As a consequence, Latinos—particularly foreign-born Latinos—are at greater risk of delayed HIV diagnosis compared with non-Latino Whites [63]. Immigrant Latinos are also at greater risk of late presentation into care than non-immigrants [63]. In addition, although AIDS survival for Latinos compared with non-Latinos is similar, foreign-born Latinos experience shorter survival compared with U.S.-born individuals [63]. This finding reinforces the need for smoking cessation treatment among Latinos living with HIV that takes into account sociocultural factors.

The findings in this study provide important insights to facilitators of smoking cessation among Latinos living with HIV. As noted by one participant, the first medical appointment to receive HIV treatment should be used as a platform to initiate smoking cessation treatment among Latino smokers. This perspective is consistent with research conducted by Vidrine et al. that has concluded that HIV treatment initiation appears to be associated with increases in intention to quit smoking, thus serving as a potential teachable moment for smoking cessation [64]. Another unique facilitator of smoking cessation among Latinos living with HIV is the existence of the AIDS Drug Assistance Program (ADAP), which is available to PLWH regardless of their immigration status [65]. In most states, nicotine replacement therapies (e.g., gum, lozenges, and patch) as well as varenicline, have been added to ADAP for treatment of tobacco use disorder [65]. This comprehensive resource increases the feasibility and reach of smoking cessation treatment among Latinos living with HIV. CBOs and clinics serving PLWH should continue to capitalize on this unique facilitator. Lastly, as noted by all participants, CBOs and clinics serving PLWH have broad outreach efforts to the Latino community (e.g., Latino nights at nightclubs, faith-based organizations, community colleges, and door-to-door outreach in counties with high concentration of Latinos). Future studies can build upon this community-based approach to conduct tobacco control research among Latino smokers living with HIV.

### Strengths and Limitations

One of the strengths of this study is the use of the SEM to provide a comprehensive view of smoking cessation among Latinos living with HIV. This approach has been recommended for PLWH elsewhere [66,67]. Another strength of this study is the participation of key leaders with different professional perspectives: medical doctors, nurse practitioners, physician assistants, case managers, research coordinators, and managers. Increasing the involvement of these professionals in supporting smoking cessation among Latinos living with HIV is a further area for future research. The inclusion of Spanish speaking participants is another study strength. However, the current study has several methodological limitations. First, all data are perceived by the key leaders of CBOs and clinics. Future research should include Latino smokers living with HIV to understand their reality and better meet the needs of this particular population. Moreover, data are self-reported and may be subject to recall bias (e.g., details of a particular Latino client and not all Latino clients). Second, there is also a possibility that participants felt compelled to offer socially desirable responses. Third, recruitment was conducted in the states of New Jersey and New York, thus limiting the emergence of other community and policy level barriers or facilitators. Fourth, the study had a small sample size. However, 10 is an acceptable number of participants for a qualitative study. The current sample size gave us enough information to understand barriers and facilitators of smoking cessation among Latinos living with HIV from the perspectives of key leaders of CBOs and clinics.

## 5. Conclusions

This qualitative study with key leaders of CBOs and clinics serving PLWH found a number of important barriers and facilitators of smoking cessation among Latinos living with HIV. In particular, it illustrates the urgent need to develop or adapt existing smoking cessation interventions to better meet the needs of this unique population. These results provide concrete operational strategies to address smoking disparities among Latino smokers living with HIV. Further research is needed on how best to integrate these perspectives into effective smoking cessation interventions.

## Figures and Tables

**Table 1:** Examples of the interview questions using the social ecological model.

Level	Examples of Interview Questions
Individual	What barriers do Latino smokers living with HIV face to quit smoking?
Interpersonal	Among Latino smokers living with HIV, what is the role of their family and friends in quitting smoking?
Organizational	What does your organization do to help Latino smokers living with HIV quit smoking?
Community	How could the community at large better support Latino smokers living with HIV to quit smoking?
Policy	What laws or policies are in effect to make it easier or more affordable for Latino smokers living with HIV to quit smoking?

**Table 2:** Participants’ type of organization and years of experience working with people living with HIV (PLWH).

Participant	Type of Organization Where They Work	Years of Experience Working with PLWH
Participant 1	Community-based organization	5 years or less
Participant 2	Community-based organization	Over 20 years
Participant 3	Health clinic	Over 20 years
Participant 4	Health clinic	Between 10 and 20 years
Participant 5	Health clinic	5 years or less
Participant 6	Community-based organization	5 years or less
Participant 7	Health clinic	Over 20 years
Participant 8	Health clinic	Between 10 and 20 years
Participant 9	Community-based organization	Between 10 and 20 years
Participant 10	Community-based organization	5 years or less

**Table 3:** Baseline characteristics of participants (*n* = 10).

Characteristics	Statistics
Age, Mean (SD)	42.3 (11.4)
Gender, *n* (%)	
Female	2 (20%)
Male	7 (70%)
Transgender	1 (10%)
Race, *n* (%)	
White	4 (40%)
Black or African American	3 (30%)
Native Hawaiian or Other Pacific Islander	1 (10%)
Other	2 (20%)
Ethnicity, *n* (%)	
Hispanic or Latino	4 (40%)
Not Hispanic or Latino	6 (60%)
Language of Preference, *n* (%)	
English	8 (80%)
Spanish	2 (20%)
Current Position, *n* (%)	
Medical Doctor	2 (20%)
Nurse Practitioner	1 (10%)
Physician Assistant	1 (10%)
Case Manager	3 (30%)
Research Coordinator	1 (10%)
Manager	2 (20%)
Manager	
Years of Experience Working with PLWH, Mean (SD)	15.1 (11.2)
Number of Clients Their Organization Serve Per Month, Mean (SD; Range)	535 (745.7; 50–2500)
Percentage of Latino Clients, Mean (SD)	33 (11.8)

## Data Availability

The datasets generated for this study are available on request to the corresponding author.
